# High Hepatitis B Virus Load in a Patient with Severe Polyarthritis Nodosa

**Published:** 2010-12-01

**Authors:** Gholam Ali Ghorbani, Gholam Hossein Alishiri, Haddi Khajeh Pour

**Affiliations:** 1 Department of Infectious Diseases, Baqiyatallah Research Center for Gastroenterology and Liver Disease, Baqiyatallah University of Medical Sciences, Tehran, Iran; 2 Department of Internal Medicine, Rheumatology Ward, Faculty of Medical Sciences, Baqiyatallah University of Medical Sciences, Tehran, Iran; 3 Department of Internal Medicine, Baqiyatallah University of Medical Science, Tehran, Iran

**Keywords:** Hepatitis B virus, Polyarteritis nodosa, Antiviral, Immunosuppressive

## Abstract

One of the extra-hepatic manifestations of hepatitis B virus is polyarteritis nodosa (PAN). It may involve medium- and small-sized arteries in any organ. Concurrency of these two diseases may be life threatening and both should be treated. Herein, we report on a patient with severe PAN and high hepatitis B virus load. The patient was an 18-year-old boy with multiple progressive wounds in the skin, referred to our center. The preliminary evaluation showed vasculitis in the skin biopsy compatible with PAN. He was treated with low dose prednisolone and lamivudine for three years. However, his condition got worse and ulcers on his leg became life threatening. The viral load was 17,000,000 copy/mL. The wound developed superimposed resistant bacterial infection. The patient was then treated with two antiviral drugs-lamivudin 100 mg/day plus adefovir 10 mg/day-and high dose cyclophosphamide (750 mg, once a month) and prednisolone (60 mg/day for one month). After six months of treatment, viral load decreased to 100,000 copy/mL and wounds healed. We concluded that high viral load of hepatitis B virus may play an important role in the severity of PAN. We recommend combination therapy with two antiviral agents with high dose of immunosuppressive drugs until both the diseases resolve significantly.

## Introduction

One of the extra-hepatic manifestations of hepatitis B virus is polyarteritis nodosa (PAN). PAN is a rare necrotizing vasculitis that can be progressive and fatal. It may be accompanied hepatitis B or C viral infection [1]. Each of the hepatitis B (HBV), C (HCV) and A viruses (HAV) can cause extra-hepatic manifestations such as PAN, nephritis and meningo-encephalitis that though may be rare, increase the mortality rate [2]. HBV infection is important in the pathogenesis of PAN and accounted for one-third of the cases with PAN, although higher prevalence rates have been reported from areas with endemic HBV infection. In Iran, HBV infection is endemic and thus, PAN is also more common than other countries [3]. Importantly, the frequency of HBV-PAN decreased following improvement in blood safety measures and vaccination campaigns [4][5] which have prolonged protection against the virus and consequently reduced its extra-hepatic complications [6]. Immune-mediated responses to HBV infection can lead to its extra-hepatic manifestations that are sometimes life threatening [7]. Load of HBV may have affect the severity of vasculitis; treating of HBV and decreasing viral load can help in recovery from vasculitis [8]. Herein, we report on a patient presented with severe PAN who had high viral load that recovered with aggressive antiviral and immunosuppressive therapy.

## Case Report

**Figure 1:**
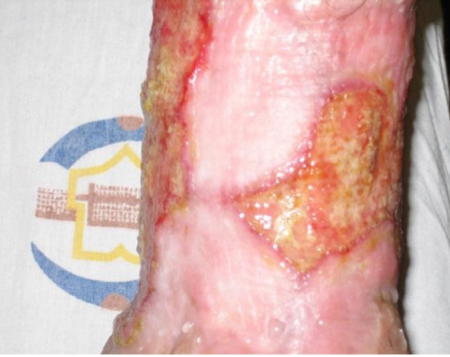
Ulcers on extremities

An 18-year-old boy was refereed to our hospital with fatigue, malaise, and the skin lesions including multiple chronic wounds on his feet and elbows and other parts of his body (Figure 1). Wounds began three years before and progressed to severe ulcers. The preliminary evaluation revealed leucocytoclastic vasculitis in the skin biopsy. HBs Ag and HBe Ag were positive but serum ALT and AST were normal. The patient had been treated with lamivudine (100 mg/day) and 15 mg/day prednisolone for the last three years. There was no response to the treatment and lesions progressed to muscle necrosis and even invaded bone so that he became candidate for below knee amputation, as there was circumferential necrotizing lesions and cyanotic ulcers in his leg. In re-evaluation for his wounds, smear and culture for leishmania, tuberculosis, fungi and malignancy were found negative. He had chronic skin lesions of molluscum contagiosum which were not active. He also had inactive ocular toxoplasmosis that had not any complications nor require any therapy. The patient had depression for his chronic multiple ulcers and the probability of leg amputation if his ulcers would not have responded to therapy.

**Figure 2:**
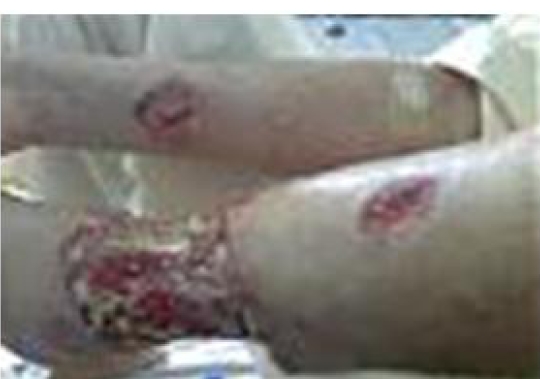
Leg wounds healed after therapy

Laboratory tests included a WBC of 10,000/mm3, ESR of 60 mm/h, and normal serum ALT, AST, ALP, and LDH. PPD skin test was negative. LE cell, FANA, cANCA, Anti-dsDNA, antiphospholipid and complements were normal. Anti-Hbs Ag and Hbc Ab were positive; Antibody against HIV and HCV were negative. HBV DNA as detected by PCR was found positive and revealed a viral load of more than 17,000,000 copy/mL using Amplicor test. New biopsy of the wounds showed necrotizing vasculitis compatible with PAN. Over the recent months, the lesions got worse with superimposed infection with multiple antibacterial resistant Klebsiella, Actinobacter and Staphylococcus aureus, and therefore, broad spectrum antibiotics were administered. Infection was eradicated with vancomycin 1 g, bid plus cefepime 1 g, bid given parenterally for one month followed by ciprofloxacin 500 mg, bid plus cefixime 400 mg, qd orally for four months. Anti-viral therapy was started with lamivudine plus adefovir in addition to immunosuppressive drugs, cyclophsphamide 1 g a month and high dose prednisolone (60 mg/day) for more than six months when wounds healed (Figure 2) and the viral load decreased to 100,000 copy/mL.

## Discussion

In this report, the extra-hepatic manifestations of HBV infection was multiple cutaneous ulcers on extremities and face. The ulcers on the leg were prone to necrosis, life threatening and did not recover with low dose prednisolone (15 mg/day) and lamivudine. Therefore, for the prevention of amputation of his leg, we started an aggressive therapy with pulse of steroid (methylprednosilone 500 mg/day) for three days followed by prednisolone 60 mg plus cyclophsphamide 750 mg stat and then each month, and also added adefovir 10 mg/day to lamivudine. After six months with this therapy, the cutaneous ulcers healed and his leg ulcers recovered. It has been suggested that incomplete therapy may delay improvement of PAN in association with HBV and the patient's life may be in danger [7][8].

High viral load may be resistant to the conventional antiviral monotherapy commonly used for hepatitis B infection, and this is why combination therapy with at least two antiviral drugs should be started as soon as possible as we did [9]. Hepatitis B monotherapy with lamivudine may increase the likelihood of resistance to this drug in long run. Therefore, drug resistance of HBV should be considered if therapy fails [8]. Low dose prednisolone cannot prevent progression of severe form of PAN as was the case in our patient who ultimately required high dose immunosuppressive therapy [9]. Viral load may be an important predictor for the severity of PAN because when the viral load decreased from 17,000,000 to 100,000 copy/mL after therapy, the patient's condition got better. Superimposed bacterial infection on ulcers can worsen the prognosis, cause seeding of bacteria in others organs and the patient is apt to develop sepsis. Therefore, superimposed bacterial infection should also be treated aggressively by appropriate antibiotics and/or surgical debridement [10]. Peginterferon and plasmapheresis are optimal therapeutic modalities; however, we treated our patient with high doses of antiviral and immunosuppressives and achieved a desirable response [11].

In patients who are given high dose immunosuppressive drugs, some latent infectious diseases should be considered and be activated. Such infections include tuberculosis, toxoplasmosis and molluscum contagiosum which should be treated if become active. All differential diagnoses of PAN should also be considered for planning the treatment [12]. Cutaneous leishmaniasis is one of the differential diagnoses for cutaneous PAN, especially in endemic areas like Iran because chronic small skin ulcers of PAN resemble leishmaniasis lesions. While immunosuppressive therapy is good for the former condition, it may disseminate the latter. Fortunately, in our patient smear and culture for leishmania were negative [13]. Toxoplasma gondii is a ubiquitous protozoan parasite causing severe or life threatening infections in immunocompromised patients. Occult ocular toxoplasmosis may be activated in patients who take high dose and prolonged immunosuppressive therapy. Toxoplasmosis is also endemic in our country. Our patient though had inactive ocular toxoplasmosis, did not get the active form of the disease during follow-up, hence no therapy was required [14].

Tuberculosis can involve the skin and create cutaneous wounds resembling PAN. Immunosuppressive therapy may activate latent tuberculosis. Fortunately, our patient had negative PPD skin test and did not need any anti-tuberculosis therapy. In endemic areas for tuberculosis, immunosuppressive therapy should be used with caution [15]. Molluscum contagiosum is a common self-limited skin condition. However, it may be disseminated in immunosuppressive patients or present as atypical lesions, although some of the antiviral drugs such as cidofovir successfully treated severe forms of molluscum contagiosum. In our patient, adefovir may have prevented activation of molluscum contagiosum [16]. Some malignant diseases may have presentations in the skin and should be considered in the differential diagnosis for PAN which in our patient was found negative [17]. Pyoderma gangrenosum is another condition that resembles the skin lesions to be differentiated from PAN. Its pathogenesis is not known but it may be a hyper-reactive phenomenon. Pyoderma gangrenosum can cause chronic skin wounds that rarely need steroid therapy except in severe form. However, in PAN, the small- and middle-sized arteries are involved that causes systemic vasculitis and steroid therapy is often needed [18]. In 30% of patients with PAN, HBV infection is present, but no infection is seen in pyoderma gangrenosum [8][19].

Leukocytoclastic vasculitis is another disease to be differentiated from PAN. This vasculitis is usually seen in inflammatory bowel disease without any relation to HBV infection. This differential diagnosis is very important since antiviral therapy is needed for PAN related to HBV [20]. Chronic diseases in any patient can cause disappointment and depression with resultant reduction in the quality of life such as what we observed in our patient. Therefore, this problem also should be considered and treated appropriately in a timely manner [21].
